# Sanggenon C Ameliorates Cerebral Ischemia-Reperfusion Injury by Inhibiting Inflammation and Oxidative Stress through Regulating RhoA-ROCK Signaling

**DOI:** 10.1007/s10753-020-01225-w

**Published:** 2020-04-02

**Authors:** Yilei Zhao, Jingfeng Xu

**Affiliations:** Department of Radiology, The First Affiliated Hospital, Zhejiang University School of Medicine, No. 79, Qingchun Road, Hangzhou, 310003 Zhejiang China

**Keywords:** ischemia, sanggenon C, inflammation, oxidative response, RhoA-ROCK signaling

## Abstract

Sanggenon C (SC), a natural flavonoid extracted from Cortex Mori (*Sang Bai Pi*), is reported to possess anti-inflammatory and antioxidant properties in hypoxia. The present study aimed to investigate the therapeutic potential and the underlying mechanisms of SC in cerebral ischemia-reperfusion (I/R) injury. A rat model of reversible middle cerebral artery occlusion (MCAO) was used to induce cerebral I/R injury *in vivo*, and SC was administrated intragastrically. Brain injuries were evaluated using Bederson scores, brain water content, and 2, 3, 5-triphenyltetrazolium chloride (TTC) staining. The levels of inflammatory factors and oxidative stress were examined using corresponding kits. Cell apoptosis was evaluated by TUNEL. Moreover, the expressions of apoptosis-related and RhoA/ROCK signaling-related proteins were detected through western blotting. *In vitro*, RhoA was overexpressed in oxygen-glucose deprivation and reperfusion (OGD/R)-induced PC12 cells to confirm the contribution of RhoA-ROCK signaling inhibition by SC to the neuroprotective effects post OGD/R. Pretreatment with SC significantly ameliorated the neurologic impairment, brain edema, and cerebral infarction post MCAO-reperfusion, associated with reductions of inflammation, oxidative stress, and cell apoptosis in the brain. Furthermore, SC remarkably downregulated the expression of RhoA/ROCK signaling-related proteins post MCAO-reperfusion in rats, while overexpression of RhoA reversed the beneficial effects of SC on protecting against inflammation and oxidative stress in OGD/R-induced PC12 cells. Taken together, these findings demonstrated that SC exerts neuroprotective effects after cerebral I/R injury *via* inhibiting inflammation and oxidative stress through regulating RhoA-ROCK signaling, suggesting a therapeutic potential of SC in cerebral I/R injury.

## INTRODUCTION

Stroke is a leading cause of millions of deaths and permanent disabilities worldwide [4]. Ischemic stroke is the most common subset of strokes and accounts for the majority of stroke-induced injuries. When ischemia occurs, blood supply to the brain will be partly interrupted as a result of thrombosis, embolism. or hypoperfusion [30]. Although vessel recanalization can be commonly achieved with treatments in clinic, it does not necessarily bring good clinical outcomes, since ischemia-reperfusion process usually leads to long-term or even irreversible injuries in the brain [17, 20]. Especially, ischemic stroke elicits strong neuroinflammatory and oxidative responses post-perfusion, resulting in neuronal excitotoxicity and apoptosis.

Sanggenon C (SC) is a flavonoid ingredient enriched in Cortex Mori, a Chinese herb also named *Sang Bai Pi*, and traditionally used for treating anti-inflammation, analgesia, and blood stasis dissipation [3]. It has been well-reported that SC can decrease the levels of proinflammatory factors, reactive oxygen species (ROS), and cell apoptosis under hypoxia, a pathological condition which shares comparative characteristics with ischemic stroke [9, 14]. However, whether and how SC is effective in ameliorating neuroinflammation and oxidative stress induced by cerebral ischemia-reperfusion remain to be elucidated.

It has been well-documented that inflammatory responses and oxidative stress post-ischemic are regulated by RhoA (Ras homolog gene family, member A), a small GTPase protein in the Rho family [7]. The activation of RhoA and its downstream effectors, Rho-dependent coiled-coil kinases (ROCK), exacerbates neuroinflammation and cytotoxicity by inducing the phosphorylation of LIM kinase (LIMK) and actin-depolymerizing factor cofilin (CFL) [16, 25]. SC was reported to exert inhibitory effects on calcineurin-NFAT2 signaling, a proinflammatory cascade reported to be activated in the ischemic-reperfusion [13, 27]. Since calcineurin-NFAT2 signaling is a downstream target of the RhoA-ROCK cascade, we hypothesized that SC might exert anti-neuroinflammatory and neuroprotective effects through inhibiting RhoA-ROCK signaling in the brain post ischemia-reperfusion.

In the current study, the effects of SC on the middle cerebral artery occlusion (MCAO)-reperfusion rats were investigated, and the potential mechanisms were explored by RhoA overexpression in oxygen-glucose deprivation and reperfusion (OGD/R)-induced PC12 cell model. Collectively, our data here emphasized the neuroprotective effects of SC on ischemic stroke through inhibiting inflammation and oxidative stress through regulating RhoA-ROCK signaling pathway.

## MATERIALS AND METHODS

### Animals

A total of sixty SPF grade adult male Sprague-Dawley (SD) rats (200–250 g) were purchased from Shanghai SLAC Laboratory Animal Company Ltd. (Shanghai, China). All animals were housed in individually ventilated cages (IVC) (*n* = 2 in each cage) under standard conditions with 12 h alternating light/dark cycle. Rats were given free access to water and standard rat chow. All of the experiment protocols were approved by the Animal Care and Use Committee of the First Affiliated Hospital, Zhejiang University School of Medicine.

### Establishment of MCAO-Reperfusion Model and Drug Administration

Rats were divided into six groups randomly (*n* = 10 in each group): control, MCAO (model group), Nimodipine (positive control group), and SC low-, middle-, and high-dose groups (1, 10, and 100 mg/kg). Animals in the SC groups were administrated intragastrically with SC (Chengdu Mansite Biotech Co., Ltd.; Chengdu, China) everyday, consecutively for a week. Saline was used as vehicle, and Nimodipine was administrated instead of SC in the positive control group. Reversible MCAO surgery was performed 1 h after the last administration of drugs or saline, using an improved Longa-Zea method as previously described [15, 26]. In brief, rats were anesthetized with 50 mg/kg pentobarbital sodium and then fixed in a supine position. Both the proximal ends of the common carotid artery (CCA) and the external carotid artery (ECA) were ligated, and the internal carotid artery (ICA) was clamped temporarily. A V-shaped oblique incision was made at the bifurcation of ECA and ICA with vascular scissors. Reopening the artery clamp, while inserting a paraffin bolt pasting through the ECA stump into the ICA until a slight resistance was felt (for a total distance about 2 cm). The time was set as the beginning of embolism. The upper end of the CCA was then ligated, and the wound was sewn up. Finally, the paraffin bolt was gently pulled back to the incision of ECA 90 min after embolism to get the reperfusion, and ischemia-reperfusion injuries were evaluated 24 h later.

### Bederson Behavioral Assessment

Neurologic symptoms post ischemia-reperfusion were assessed using the Bederson scale as described in the previous study [1]. Briefly, behavioral scores of rats were evaluated based on parameters involved in flexion, lateral push, and circling, varying from 0 to 3: 0, no detectable neurological symptom; 1, any deficits in forelimb stretching; 2, forelimb flexion, consistent reduction in resistance to lateral push toward paretic side; and 3, forelimb flexion, resistance reduction, and consistent circling [5]. All assessments were performed by an experienced experimenter with no knowledge about animals’ grouping.

### Brain Wet-Dry Weight Ratio

Brains were removed 24 h after MCAO-reperfusion and washed with saline. Excess water was sucked up with clean filter paper. The wet and dry weights of rat brains were examined before and after stoving in 55 °C until a constant weight respectively, using an electronic balance (AR1140, OHAUS, USA). The ratio of the wet-dry weight ratio (W/D) was calculated.

### 2, 3, 5-Triphenyltetrazolium Chloride Staining

The degree of cerebral infarction was evaluated using 2, 3, 5-triphenyltetrazolium chloride (TTC) staining. Brains were washed with saline 24 h post ischemia-reperfusion and removed rapidly on ice and sliced into six coronal sections (2-mm thick). These sections were immersed in 1% TTC (Sigma-Aldrich, San Jose, CA, USA) and subsequently fixed in 4% paraformaldehyde until imaging. The presence or absence of infarction was determined by examining the areas stained with or without TTC, respectively. The area of cerebral infarction was qualified using the ImageJ software (National Institutes of Health, Bethesda, MA, USA), and data were normalized to the nonischemic brain and expressed as a percentage.

### Measurement of Inflammatory Factors

Rat’s blood was collected 24 h post ischemic-reperfusion. The levels of tumor necrosis factor-alpha (TNF-α), interleukin 1-beta (IL-1β), and interleukin-6 (IL-6) in serum and the culture supernatant of PC12 cells were detected using enzyme-linked immunosorbent assay (ELISA) kits on the basis of the manufacturer’s protocols. The abovementioned kits were obtained from Shanghai Xitang Biotechnology Co., Ltd. (Shanghai, China).

### Determination of Oxidative Stress-Related Markers

The content of ROS and malondialdehyde (MDA) and activity of superoxide dismutase (SOD) in the tissue homogenate or cells were determined using commercial kits (Nanjing Jiancheng Bioengineering Institute; Nanjing, China) according to the colorimetric methods.

### Terminal Deoxynucleotidyl Transferase dUTP Nick End Labeling

The transferase dUTP nick end labeling (TUNEL) staining was performed to evaluate cell apoptosis post ischemia-reperfusion in the hippocampus using a commercial kit labeling DNA strand breaks with FITC (Beyotime, China) in accordance with the manufacturer’s guidelines. The stained sections were detected using a confocal laser scanning microscope. For apoptosis of PC12, cells were fixed with 4% paraformaldehyde after washing with phosphate-buffered saline. The TUNEL staining (Beyotime, China) was utilized to visualize the apoptotic cells. The nuclei of healthy cells were stained blue, whereas apoptotic cells with nuclei presented brown/yellow staining were identified as TUNEL-positive cells.

### Western Blotting

The hippocampus tissues and PC12 cells were homogenized with RIPA lysis buffer and then centrifuged to obtain the supernatant. Total proteins were extracted using RIPA lysis buffer (Beyotime, Shanghai, China). The protein concentration was detected using a bicinchoninic acid (BCA) protein assay kit (Beyotime, Shanghai, China). Proteins were separated in SDS-PAGE gel and then transferred onto polyvinylidene fluoride (PDVF) membranes (Merck Millipore). The membranes were blocked with 5% non-milk and then incubated with primary antibodies (Cell Signaling Technology, Boston, MA, USA) at 4 °C overnight. Following incubation with secondary antibodies (Stanta Cruz Biotechnology, CA, USA), protein bands were detected with an enhanced chemiluminescence kit (Thermo Scientific, USA). Intensities of bands were detected by using the ImageJ software (National Institutes of Health, Bethesda, MA, USA). The protein expression was normalized to GAPDH levels.

### Cell Culture and Treatment

PC12 cell was provided by the Culture Collection of Chinese Academy of Science (Shanghai, China). Cells were maintained in Dulbecco’s modified Eagle’s medium (DMEM) (Gibco) containing 10% fetal bovine serum (Gibco) under a concentration of 5% CO_2_. The culture medium was replaced every 2 days. PC12 cells were pretreated with SC (1, 10, and 100 μM) for 12 h before OGD/R or normoxic manipulations. For transfection, plasmids used for RhoA overexpression were constructed by GenePharma (Shanghai, China), and empty plasmid carrying no RhoA pcDNA was used as control. The transfection was performed using Lipofectamine 2000 reagent (Invitrogen) following the manufacturer’s recommendations.

### Establishment of Cell OGD/R Model

Cells were plated in 95-cm cell culture dish (1 × 10^6^ cells/well) and incubated at 37 °C. Cells in the logarithmic growth phase were cultured in glucose-free DMEM and placed in an anaerobic chamber (Thermo scientific, Waltham, USA) under a gas mixture of 1% O_2_, 94% N_2_, and 5% CO_2_ for 2 h. OGD was terminated by restoring with glucose at DMEM and incubated under normoxic conditions (95% air, 5% CO_2_) for 24 h. In the control group, cells were incubated under normoxic conditions all the time.

### Cell Viability Assay

Following treatment, cell viability was evaluated using the cell counting kit-8 (CCK-8) assay (Dojindo Laboratories, Kumamoto, Japan). Cell suspension was dispensed into a 96-well plate (5000 cells/well), which was pre-incubated for 24 h in a humidified incubator at 37 °C under 5% CO_2_. A total of 10 μL CCK-8 solution was added into each well of the plate. The absorbance at 450 nm was determined using a microplate reader.

### Statistical Analysis

All experiments were performed with at least independent three replicates. Data were presented as means ± standard deviation (SD). All data were analyzed and plotted using GraphPad prism version 6.0 (GraphPad Software, Inc.). Comparisons between the two groups were conducted using two-tailed Student’s *t* test. One-way analysis of variance (ANOVA) followed by the use of Tukey’s test to compare multiple groups. *P <* 0.05 was considered to indicate a statistically significant difference.

## RESULTS

### SC Ameliorated Neurological Injuries Post MCAO-Reperfusion

**Fig. 1:**
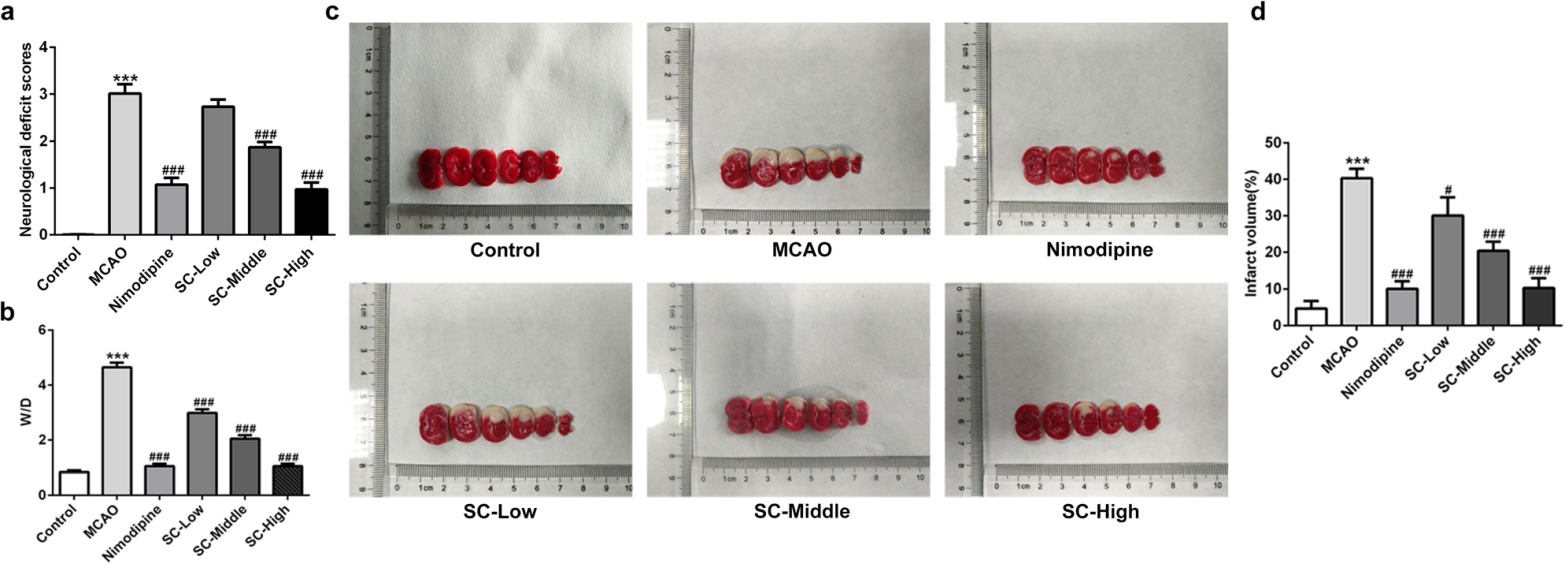
SC ameliorated neurological injuries post MCAO-reperfusion. **a** Neurologic symptoms post ischemia-reperfusion were assessed using the Bederson scale. **b** The ratio of W/D was calculated. **c** The volume of the cerebral infarction was assessed by TTC staining in each group. **d** The percentage of infarct volume was calculated. ^***^P < 0.001 *vs.* control; ^#^*P* < 0.05, ^###^*P* < 0.001 *vs.* MCAO.

To test the potential neuroprotective effects of SC on cerebral ischemia-reperfusion injury, low, middle, and high doses of SC (1, 10, and 100 mg/kg) were administrated intragastrically into rats for a consecutive week prior to MCAO-reperfusion operations. Results from Fig. 1a indicated that MCAO-reperfusion induced remarkable neurological deficits, but SC pretreatment significantly ameliorated these deficits in a dose-dependent manner, as evaluated with the Bederson scale. In addition, prior SC, administration alleviated the brain edema post MCAO-reperfusion, as revealed by the obvious decrease in brain water content in SC-treated rats compared with rats in the MCAO group (Fig. 1b). Moreover, a 100 mg/kg dose of SC produced comparative outcomes with Nimodipine, a calcium channel blocker clinically used in the treatment of cerebral vasospasm and resultant ischemia, used as a positive control in the present study. Besides, SC treatment also dose-dependently reduced the total area of cerebral infarction induced by MCAO-reperfusion (Fig. 1c and d). Taken together, these results suggest that prior treatment of SC exerts neuroprotective effects on MCAO-reperfusion injury.

### SC Alleviated the Neuroinflammation and Oxidative Stress Post MCAO-Reperfusion

**Fig. 2:**
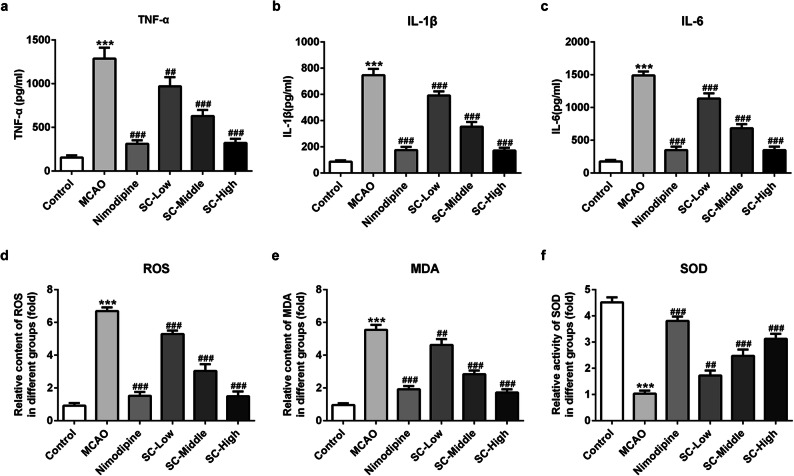
SC alleviated the neuroinflammation and oxidative stress post MCAO-reperfusion. The levels of TNF-α (**a**), IL-1β (**b**), and IL-6 (**c**) in serum of each rat were tested using ELISA. The concentrations of ROS (**d**), MDA (**e**), and the activity of SOD (**f**) in brain tissues were detected using commercial kits. ^***^P < 0.001 *vs.* control; ^##^*P* < 0.01, ^###^*P* < 0.001 *vs.* MCAO.

To investigate whether SC is effective in preventing neuroinflammation and oxidative stress, the contents of inflammation- and oxidative stress-related markers of rats in each group were detected 24 h post MCAO-reperfusion. As exhibited in Fig. 2a–c, prior administration of SC dose-dependently decreased the levels of inflammatory factors, including TNF-α, IL-1β, and IL-6. In addition, the contents of ROS and MDA were also reduced, accompanied by enhanced activity of antioxidant enzyme SOD in rats treated with SC instead of saline (Fig. 2d–f), indicating the alleviation of oxidative stress by SC post MCAO-reperfusion. These data uncover that SC can attenuate neuroinflammation and oxidative stress post MCAO-reperfusion in rats.

### SC Decreased Cell Apoptosis Induced by MCAO-Reperfusion

**Fig. 3:**
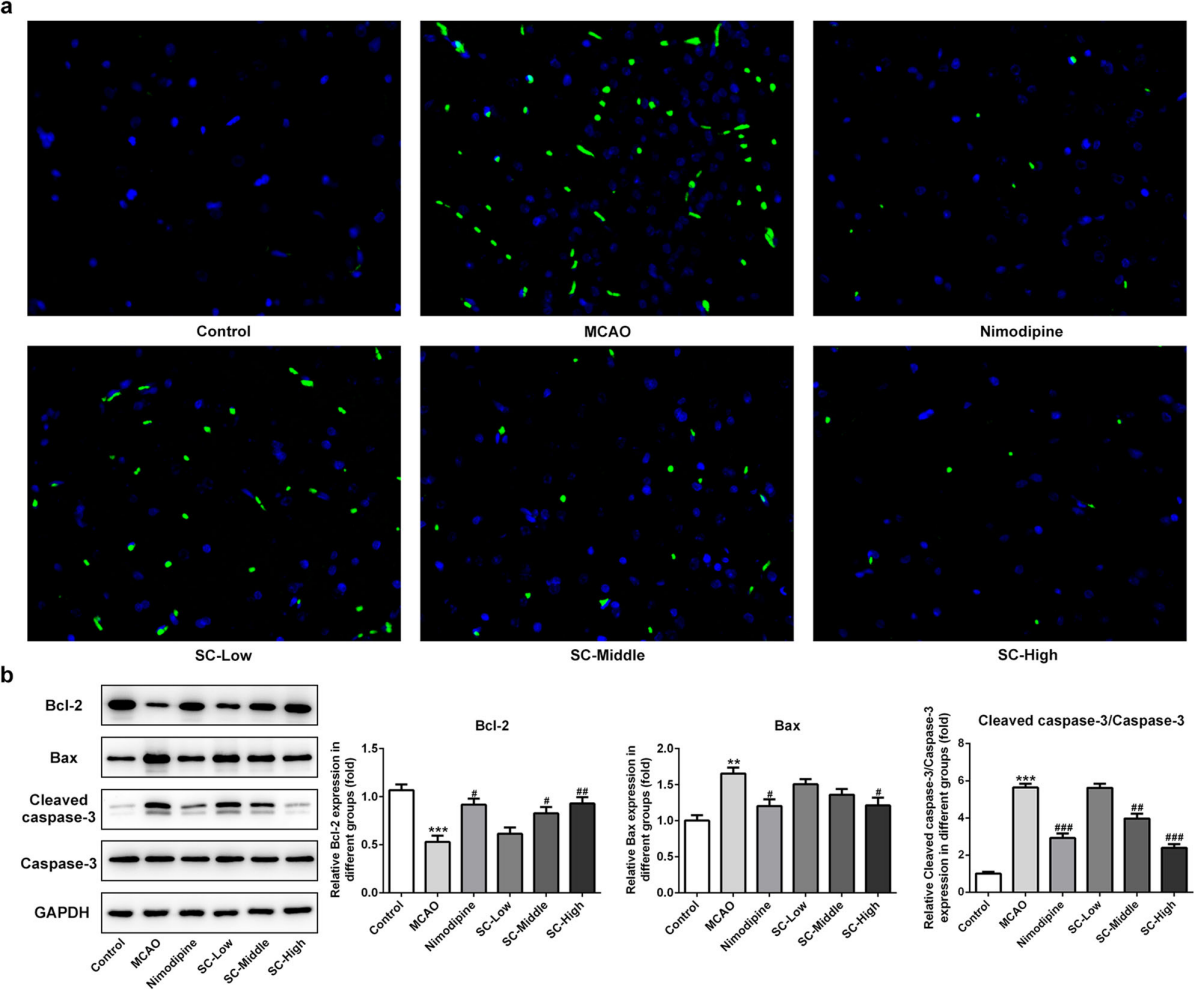
SC decreased cell apoptosis induced by MCAO-reperfusion. **a** Apoptosis of cells was examined using TUNEL assay. (magnification, × 200). **b** The expression of apoptosis-related proteins was determined using western blot analysis. ^**^P < 0.01, ^***^P < 0.001 *vs.* control; ^#^*P* < 0.05, ^##^*P* < 0.01, ^###^*P* < 0.001 *vs.* MCAO.

To explore whether SC could protect brain cells from apoptosis after MCAO-reperfusion, we performed TUNEL staining in rat brain slices that were obtained 24 h after MCAO-reperfusion. Compared with sham surgery, MCAO-reperfusion dramatically increased the number of TUNEL-positive cells in the hippocampus. However, Nimodipine and SC significantly prevented brain cells from apoptosis post MCAO-reperfusion (Fig. 3a). Furthermore, the expressions of apoptosis-related proteins were examined using western blotting. As presented in Fig. 3b, as compared with the control group, SC remarkably downregulated the expressions of Bax and cleaved caspase-3 (two pro-apoptotic proteins), but obviously upregulated the expression of Bcl-2 (an anti-apoptotic protein). These findings provided a clue that SC can suppress cell apoptosis in the brain post MCAO-reperfusion.

### SC Inhibited RhoA-ROCK Signaling Post MCAO-Reperfusion

**Fig. 4:**
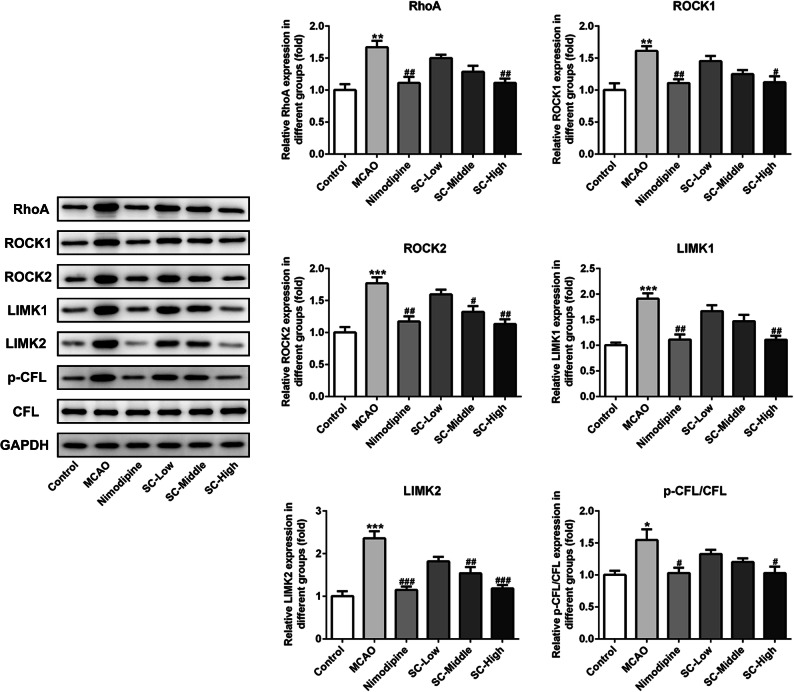
SC treatment inactivated RhoA-ROCK signaling pathway. The expression of RhoA-ROCK signaling-related proteins was measured using western blot analysis. ^*^P < 0.05, ^**^P < 0.01, ^***^P < 0.001 *vs.* control; ^#^*P* < 0.05, ^##^*P* < 0.01, ^###^*P* < 0.001 *vs.* MCAO.

RhoA-ROCK signaling activation was reported to aggravate neuroinflammation and oxidative stress in the IR injury [7]. To test whether and how the RhoA-ROCK signaling pathway was regulated by SC, we examined the expressions of several key proteins in the RhoA-ROCK signaling pathway. As shown in Fig. 4, prior treatment with SC notably downregulated the expressions of RhoA, ROCK1, and ROCK2, as well as the downstream LIMK1, LIMK2, and phosphorylated CFL in the hippocampus. These observations reveal that SC inhibits RhoA-ROCK signaling post MCAO-reperfusion.

### RhoA-ROCK Signaling Inhibition Contributed to the Cytoprotective Effects of SC in OGD/R-Induced PC12 Cells

**Fig. 5:**
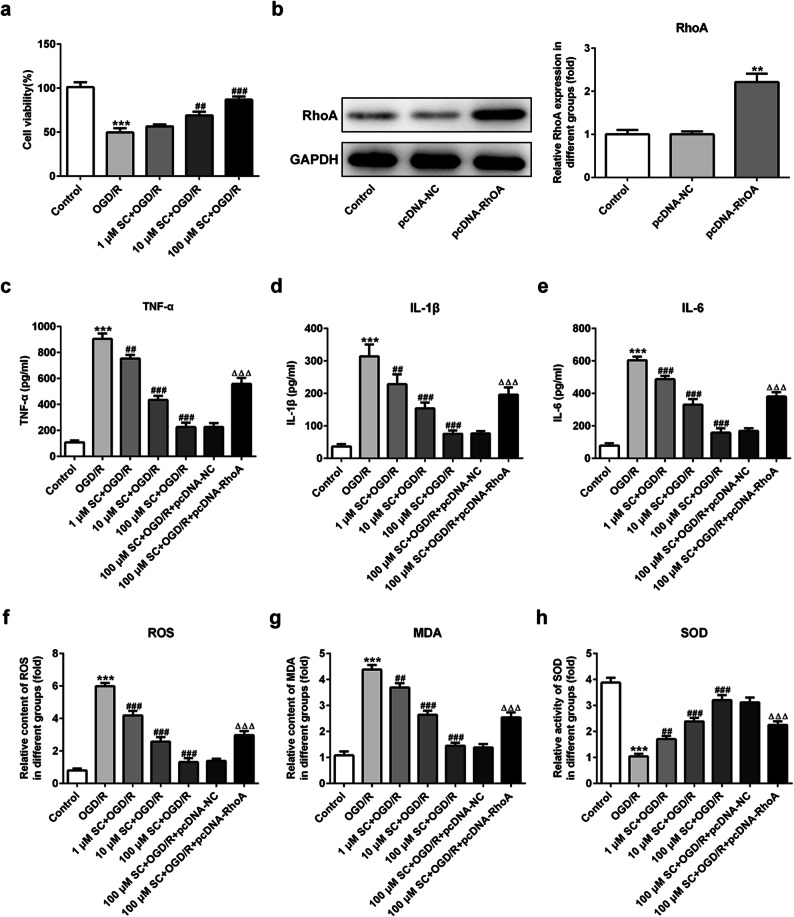
RhoA overexpression reversed the inhibitory effects of SC on inflammation and oxidative stress in OGD/R-induced PC12 cells. **a** Cell viability of PC12 cells was detected using CCK-8 assay after OGD/R induction. ^***^P < 0.001 *vs.* control; ^##^*P* < 0.01, ^###^*P* < 0.001 *vs.* OGD/R. **b** The expression of RhoA was assessed using western blotting after transfection with RhoA overexpressed plasmids. ^**^P < 0.01 *vs.* pcDNA-NC. The concentrations of **c** TNF-α, **d** IL-1β, and **e** IL-6 in the culture supernatant of PC12 cells were examined using ELISA. The contents of **f** ROS, **g** MDA, and the activity of **h** SOD in cells were determined using commercial kits. ^***^P < 0.001 *vs.* control; ^##^*P* < 0.01, ^###^*P* < 0.001 *vs.* OGD/R; ^△△△^*P* < 0.001 *vs.* 100 μM SC + OGD/R + pcDNA-NC.

**Fig. 6:**
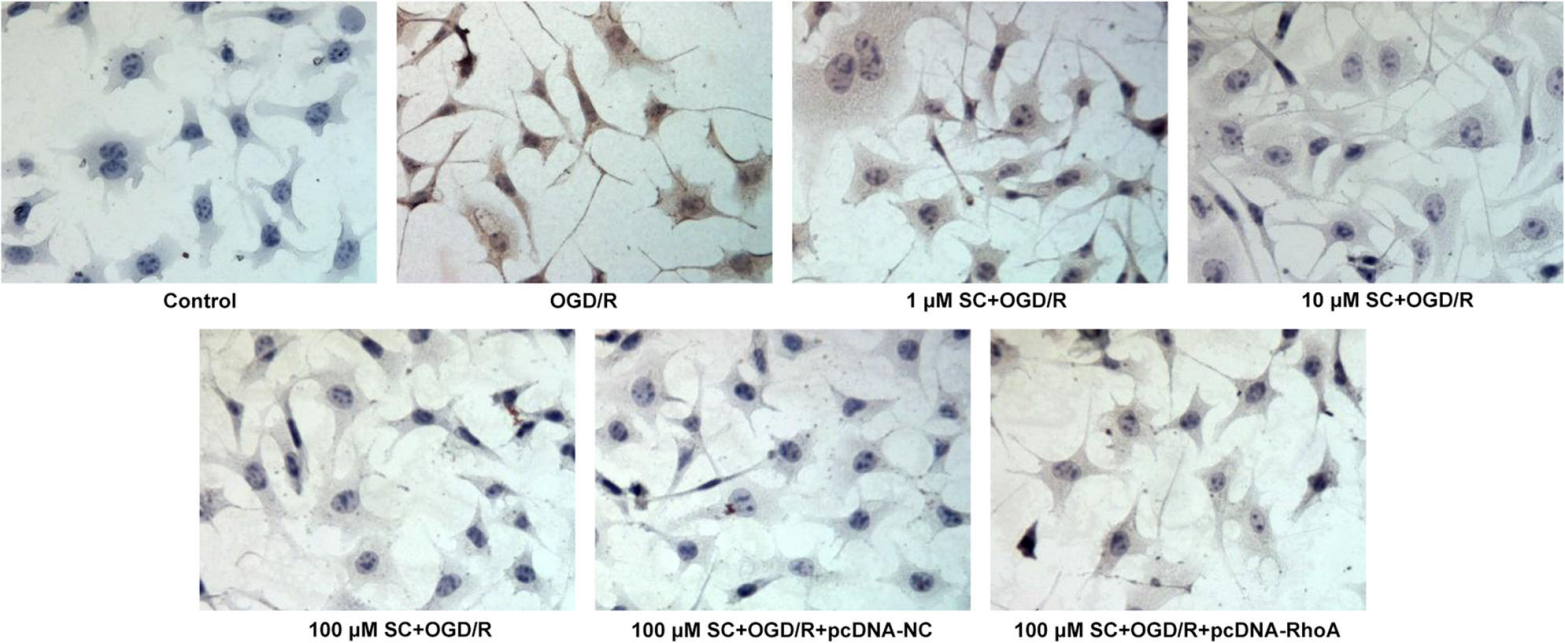
RhoA overexpression restored the inhibitory effects of SC on apoptosis in OGD/R-induced PC12 cells. Apoptosis of PC12 cells was evaluated using TUNEL assay (magnification, × 200).

**Fig. 7:**
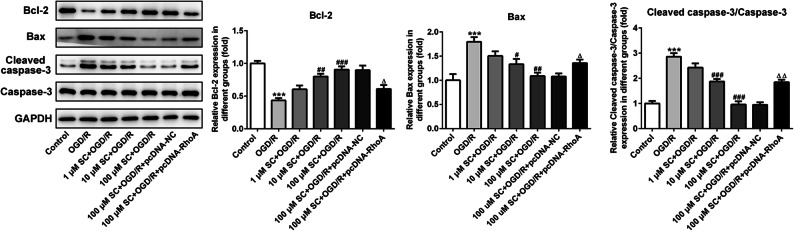
RhoA overexpression attenuated the regulatory effects of SC on apoptosis-related proteins expression in OGD/R-induced PC12 cells. Western blot analysis was employed to examine the expression of Bcl-2, Bax, and cleaved caspase-3. ^***^P < 0.001 *vs.* control; ^#^*P* < 0.05, ^##^*P* < 0.01, ^###^*P* < 0.001 *vs.* OGD/R; ^△^*P* < 0.05, ^△△^*P* < 0.01 *vs.* 100 μM SC + OGD/R + pcDNA-NC.

**Fig. 8:**
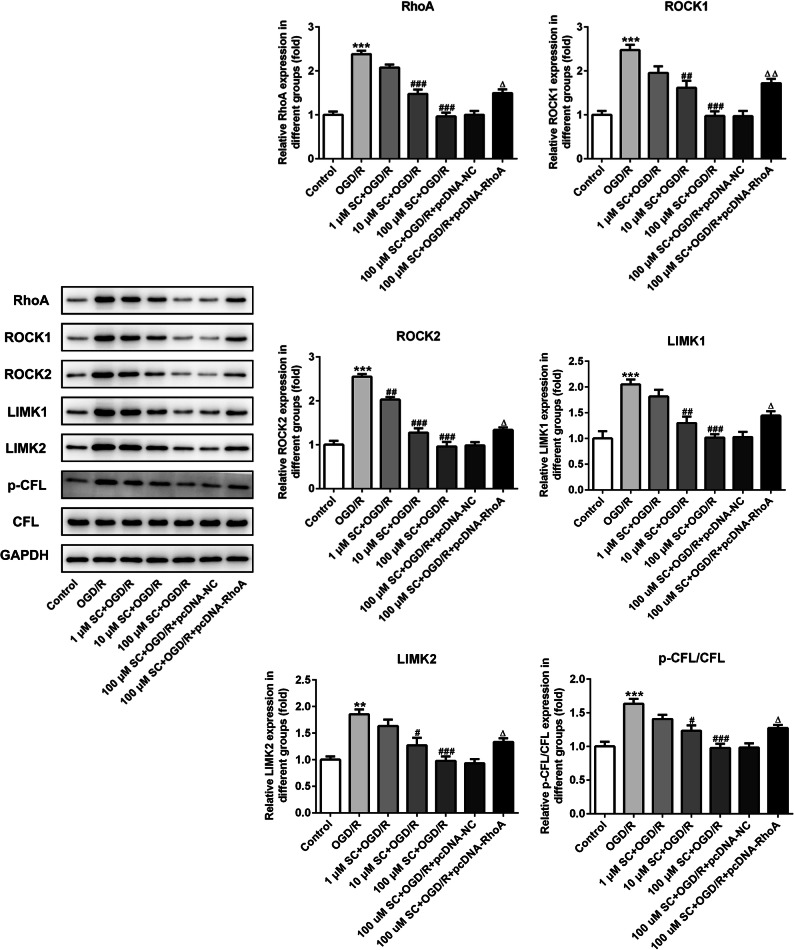
RhoA overexpression relieved the regulatory effects of SC on RhoA-ROCK signaling pathway. The expression of RhoA-ROCK signaling-related proteins was detected using western blot analysis. ^**^P < 0.01, ^***^P < 0.001 *vs.* control; ^#^*P* < 0.05, ^##^*P* < 0.01, ^###^*P* < 0.001 *vs.* OGD/R; ^△^*P* < 0.05, ^△△^*P* < 0.01 *vs.* 100 μM SC + OGD/R + pcDNA-NC.

To confirm the contribution of RhoA-ROCK signaling inhibition by SC to its neuroprotective effect on I/R, we established the OGD/R model in PC12 cells, a cell line derives from a pheochromocytoma of the rat adrenal medulla and can acquire neuron-like properties when exposed to nerve growth factor (20), to mimic the MCAO-reperfusion *in vitro* [18]. OGD/R for 24 h dramatically decreased the cell viability to 50%, which had been significantly prevented by SC in a dose-dependent manner (Fig. 5a). Subsequently, RhoA overexpression (Fig. 5b) combined with SC treatment was employed to observe the potential mechanisms. Expectedly, OGD/R aggravated inflammatory responses, oxidative stress, and cell apoptosis in PC12 cells, but all of them were significantly rescued following treatment with SC. In detail, in a dose-dependent way, SC significantly decreased the level of TNF-α, IL-1β, and IL-6 (Fig. 5c–e), reduced the contents of ROS and MDA (Fig. 5f and g), enhanced the activity of SOD (Fig. 5h), inhibited cell apoptosis coupled with the downregulation of the expressions of Bax, cleaved-caspase3 and the upregulation of Bcl-2 level (Figs. 6 and 7, and suppressed the expression of RhoA-ROCK signaling-related proteins post OGD/R (Fig. 8), which were in line with the results *in vivo*. Furthermore, RhoA overexpression hindered all the above beneficial effects of SC on the OGD/R-induced PC12 cell injury. These observations demonstrate that the inhibition of RhoA-ROCK signaling by SC contributes importantly to its anti-inflammatory, anti-oxidative, and cytoprotective effects on OGD/R injury.

## DISCUSSION

Ischemic stroke accounts for millions of disabilities and deaths worldwide [6]. Unfortunately, pharmacological recanalization does not always bring good outcomes in clinic, since reperfusion itself induces neuronal injuries that are hard to be restored in the brain [8, 23]. Neuroinflammation and oxidative stress are reported to be dramatically upregulated during the ischemia-reperfusion processes, both of which have been evidenced to play important roles in the neuronal damages in the stroke [20]. Thus, anti-inflammatory and anti-oxidative agents possess great potentials in preventing or ameliorating the neuronal injuries post ischemia-reperfusion. Several herbs, such as Honeysuckle (*Jin Yin Hua*), Radix Isatidis (*Ban Lan Gen*), and Cortex Mori (*Sang Bai Pi*), have been used as anti-inflammatory and anti-oxidative constituents in traditional Chinese medicine for thousands of years. The active ingredients of those herbs and the underlying mechanisms of their potential anti-inflammatory and neuroprotective effects on ischemic stroke deserve further investigation. In the present study, we focused on the neuroprotective effect of a main flavonoid extract from Cortex Mori SC pn cerebral ischemia-reperfusion.

A definite anti-inflammatory and anti-oxidative effect of SC had been documented in various pathological conditions [28]. For instance, SC downregulated the levels of proinflammatory factors (TNF-α, IL-1β, and IL-6), while upregulated the activities of antioxidant enzymes in cardiomyocyte hypoxia; a complication usually occurs associated with ischemic stroke [9]. Besides, treatment with SC remarkably ameliorated the cardiac hypertrophy, fibrosis, and deteriorated systolic and diastolic function induced by aortic banding through inhibiting inflammatory responses in the heart [24]. In the present study, we found that prior treatment with SC for a week was sufficient to reduce inflammatory and oxidative responses, inhibit cell apoptosis in the brain, and ameliorate cerebral infarction post ischemia-reperfusion in a dose-dependent manner.

Report has demonstrated previously that inflammation and oxidative stress are closely implicated in the induction of neurodegeneration and neuronal apoptosis [10]. Compelling evidence indicated that massive cell apoptosis is observed post ischemia-reperfusion in the brain [29]. Here, we reported that SC is effective in preventing cell death, possibly due to its anti-inflammatory and anti-oxidative effects. Indeed, SC has been reported to reduce the hypoxia-induced apoptosis as detected by decreased TUNEL staining and increased Bcl-2 the expression [9]. It is interesting that an anticancer role of SC in inducing apoptosis of cancer cells had been observed in several previous studies [11, 31]. Additionally, SC could also promote the proliferation of osteoblasts while inhibit the formation and function of osteoclasts [21]. The mechanisms underlying when and how SC switches between the pro- and anti-cell apoptosis remain elusive and deserve further investigation.

To further investigate the potential mechanisms of SC in cerebral ischemia-reperfusion, the expressions of proteins in RhoA-ROCK signaling pathway were detected. We identified that the inhibition of RhoA-ROCK signaling pathway by SC contributes to its neuroprotective effect post ischemia-reperfusion. RhoA is involved in the proinflammatory cascades and was reported to be activated in the brain areas with focal cerebral infarction [12]. Emerging evidence supports that RhoA-ROCK signaling inhibition can alleviate the neuroinflammation and post-ischemic neuronal damages [2]. Moreover, previous study has highlighted the importance of SC in anti-inflammatory effect under cardiac hypertrophy through inactivating the calcineurin-NFAT2 pathway, a downstream cascade of RhoA-ROCK signaling pathway [19, 24]. Astrocytic activation of calcineurin and NFAT, which was reported in the process of ischemia-reperfusion, was found to aggravate inflammatory responses in cerebrovascular diseases [22]. In the present study, we found a dramatic activation of RhoA-ROCK-LIMK-CFL signaling post MCAO-reperfusion and OGD/R, which can be significantly and dose-dependently reversed by the prior administration of SC. Furthermore, RhoA-overexpression significantly hindered the beneficial effects of SC on OGD/R.

Taken together, our results indicate that SC exerts anti-inflammatory and anti-oxidative effects post cerebral ischemia-reperfusion through inhibiting the RhoA-ROCK signaling. These findings evidenced a therapeutic potential of SC in the ischemic stroke.
